# High Expression of the Costimulatory Checkpoint Factor DNAM-1 by CD4^+^ T-Cells from Multiple Myeloma Patients Refractory to Daratumumab-Containing Regimens

**DOI:** 10.1007/s44228-022-00013-7

**Published:** 2022-08-09

**Authors:** Katrine Fladeland Iversen, Line Nederby, Thomas Lund, Torben Plesner

**Affiliations:** 1 Institute of Regional Health Science, University of Southern Denmark, Beriderbakken 4, 7100 Vejle, Denmark; 2 Section of Hematology, Department of Internal Medicine, Lillebaelt Hospital, University Hospital of Southern Denmark, Beriderbakken 4, 7100 Vejle, Denmark; 3 Department of Biochemistry and Immunology, Lillebaelt Hospital, University Hospital of Southern Denmark, Beriderbakken 4, 7100 Vejle, Denmark; 4 Department of Hematology, Odense University Hospital, J.B. Winsløvs Vej 4, 5000 Odense C, Denmark

**Keywords:** Multiple myeloma, Bone marrow microenvironment, Treatment, Immunotherapy, Daratumumab, Checkpoint inhibitor

## Abstract

Multiple myeloma is an incurable disease characterized by unregulated growth of malignant plasma cells in the bone marrow (BM). Tumor-induced dysfunction of T-cells may be responsible for immune evasion and failure of immunotherapy. Therefore, a better understanding of the phenotype of T-cells at the tumor site is needed. We assessed the expression of immune regulatory receptors on T-cell subsets from peripheral blood (PB) and BM using multicolor flow cytometry. Paired PB and BM samples were collected from newly diagnosed, treatment-naïve myeloma patients (*n* = 19) and patients progressing during treatment with the CD38 monoclonal antibody daratumumab alone or in combination with other anti-myeloma drugs (*n* = 39). We observed that CD4^+^ T-cells from both PB and BM of patients relapsing on daratumumab have a higher expression of the costimulatory checkpoint receptor DNAM-1. The potential role of DNAM-1^+^CD4^+^ T-cells in the development of resistance to daratumumab needs further exploration. We also observed that the inhibitory checkpoint receptor TIGIT is more frequently expressed by BM CD8^+^ T-cells from myeloma patients than PD-1 and CTLA-4, which supports the hypothesis that TIGIT may play a central role in the immune escape of the malignant plasma cells.

## Introduction

Multiple myeloma (MM) is an incurable disease characterized by uncontrolled growth of malignant plasma cells in the bone marrow (BM) [1]. As the disease advances, the tumor microenvironment in the BM becomes more permissive for tumor growth [2]. T-cell functions are impaired, possibly due to an increasing concentration of immunosuppressive adenosine in the BM microenvironment. Such impairment is reflected by higher expression of markers of T-cell exhaustion such as programmed death-1 (PD-1) and cytotoxic T-lymphocyte associated protein 4 (CTLA-4) [3–5]. Several checkpoint inhibitors that block PD-1, PD-L1 or CTLA-4 have been approved for therapy of solid cancers but, so far, checkpoint inhibitors have not shown convincing efficacy in MM [6–8]. T-cell immunoglobulin and ITIM domains (TIGIT) is an inhibitory molecule expressed by lymphocytes that competes with the activating receptor DNAX accessory molecule-1 (DNAM-1) for binding to CD155 and CD112, which is expressed by myeloma cells and other cell types [9, 10]. TIGIT is highly expressed by CD8^+^ T-cells from MM patients and may play an important, inhibitory role of the T-cell response against MM [11].

Daratumumab (DARA) is a CD38 antibody approved for treatment of MM as monotherapy or in combination with a number of standard of care anti-myeloma drugs. In addition to DARA’s ability to eradicate tumor cells directly by complement dependent cytotoxicity (CDC), antibody-dependent cellular cytotoxicity (ADCC) and antibody mediated cellular phagocytosis (ADCP), it can deplete CD38^+^ regulatory cells of T, B and monocyte origin, which leads to the expansion and activation of cytotoxic T-cells [12]. Chatterjee et al. have shown that CD38 antibodies may directly stimulate T-cell mediated anti-tumor responses, while Marlein et al. showed that CD38-dependent tumor-derived tunneling nanotubes (TNT) are established between bone marrow stromal cells (BMSCs) and MM cells. Mitochondrial transfer via these TNT is a method for the MM cell to provide energy for further tumor growth. This activity was significantly decreased by CD38 antibody in vitro [13, 14]. Additionally, DARA impairs MM cell adhesion to the BMSCs [15].

Despite the multiple modes of action of DARA, the majority of patients receiving this antibody eventually relapse [16]. The mechanisms of resistance are poorly understood. Following the first DARA infusion, there is a very significant reduction of the expression of CD38 on residual MM cells, which is associated with impaired ADCC and CDC activity by DARA [17]. This phenomenon is, however, similar in responding and non-responding patients. A high level of CD38 on MM cells before treatment is associated with a better chance of response to DARA, but it had no impact on progression free survival ([17] and Nijhof personal communication]). In other regards, a low expression of CD38 could be an advantage by preventing the formation of TNT between BMSCs and MM cells, and by preventing the adhesion of MM cells to BMSCs. CDC may also be impaired at the time of progression, due to increased expression of CD55 and CD59 by myeloma cells [17]. Whether impaired T-cell function mediated by checkpoint inhibitors plays a role in the development of resistance to DARA is not known. Therefore, we have studied the expression of inhibitory and costimulatory checkpoint molecules on T-cells isolated from peripheral blood (PB) and BM of patients progressing on a DARA-containing regimen (Daratumumab Refractory Multiple Myeloma patients; DRMM). The results were compared with the profile of treatment-naïve, newly diagnosed myeloma patients (NDMM).

## Materials and Methods

### Study Population and Sample Collection

Patients diagnosed with MM according to the IMWG guidelines at the Departments of Hematology at Vejle Hospital and Odense University Hospital were included in the study [18]. The patients were either treatment-naïve, newly diagnosed MM patients or those with progressive disease according to the IMWG criteria on a DARA-containing regimen [19]. The study was approved by the regional Ethical Committee (S-20170212). Participation was voluntary, and written informed consent was obtained from all subjects.

Samples were obtained between December 2019 and May 2021. Data on patient characteristics and prior treatment were retrospectively obtained from the electronic medical journal and, afterwards, registered in a designated Research Electronic Data Capture (REDCap) database [20, 21].

### Cell Isolation from PB and BM Aspirate

Paired PB and BM samples were obtained from NDMM (*n* = 19) and DRMM (*n* = 39). Ten milliliter of each specimen were collected immediately after each other in BD Vacutainer^®^ EDTA blood collection tubes (BD Biosciences, San Jose, CA, USA). Bone marrow mononuclear cells (BMMCs) and PB mononuclear cells (PBMCs) were isolated by density-gradient centrifugation using Ficoll-Paque™ PLUS (GE Healthcare Bio-Sciences AB. Uppsala, Sweden) according to the manufacturer’s instructions. Cells were cryopreserved in a medium of 70% RPMI 1640 with GlutaMAX™ supplement (ThermoFisher Scientific, Waltham, MA, USA), 20% heat-inactivated fetal bovine serum (FBS) (ThermoFisher Scientific), and 10% dimethyl sulfoxide (Sigma-Aldrich, St. Louis, MO, USA), and kept at − 135 °C until use. The isolation process was initiated less than 24 h after the collection.

### Flow Cytometry

Cryopreserved PBMCs and BMMCs were thawed in a 37 °C water bath and resuspended in phosphate buffered saline (PBS) with 15% heat-inactivated FBS (ThermoFisher Scientific). Concentration and viability were determined using the trypan blue exclusion method and the Countess II Automated Cell Counter (ThermoFisher Scientific). The median viability in PBMC and BMMC were 89% (range 57–98%) and 79% (range 60–90%), respectively. Cells in PBS/0.5% bovine serum albumin (BSA) were treated with Human TruStain FcX (BioLegend, San Diego, CA, USA) according to the manufacturer’s recommendations. Subsequently, monoclonal antibodies and cells were mixed in Brillant Stain Buffer Plus (BD Biosciences) and the suspensions were incubated for 15 min at room temperature. Antibodies (all from BD Biosciences) had been titrated using relevant materials: anti-TIGIT BV421 (clone 741182), anti-CD45 BV650 (clone HI-30), anti-DNAM-1 BB515 (clone DX11), anti-CD3 PerCP-Cy5.5 (clone UCHT-1), anti-CTLA-4 PE (clone BNI3), anti-PD-1 PE-Cy7 (clone EH12.1), anti-CD4 R718 (clone SK3), and anti-CD8 APC-H7 (clone SK1). Two fluorescence-minus-two (FM2) samples were prepared for each sample leaving out anti-DNAM-1 BB515 and anti-PD-1 PE-Cy7 in the first and anti-TIGIT BV421 and anti-CTLA-4 PE in the second as these showed none/minimal spectral overlap. After staining, samples were treated with 2 mL 1× BD Pharm Lyse™ Lysing buffer (BD Biosciences) and washed using PBS/0.5% BSA. Before analysis, 7-AAD (BD Biosciences) was added.

**Fig. 1:**
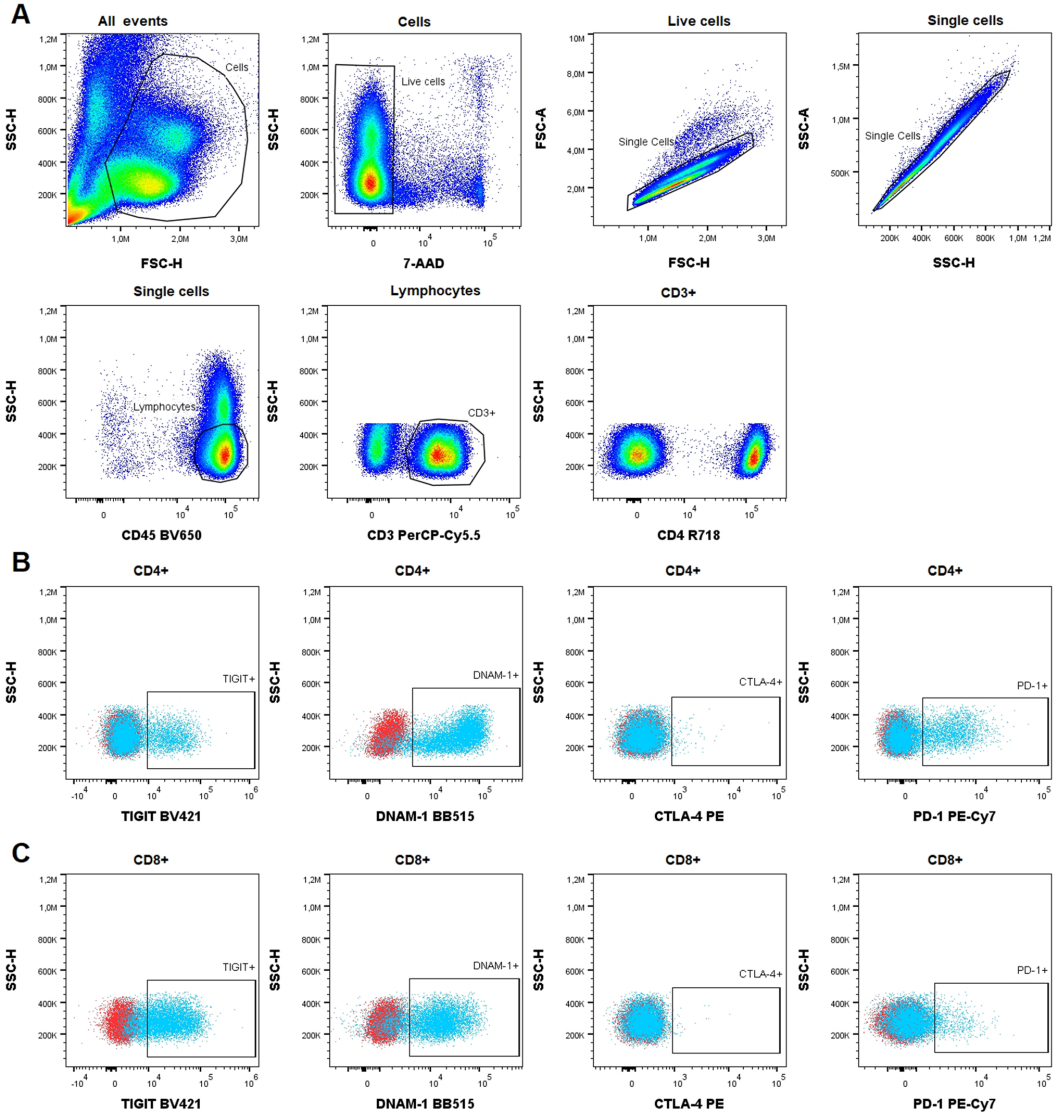
Gating strategy applied in the study shown in a representative MM patient sample. **A** In the first panel, the cell subset was defined among all events acquired in a forward scatter (FSC)–height (*H*)/Side scatter (SSC)–height (*H*) plot. Next, within this cell subset, live cells (7-AAD^−^) were selected for further analyses. In the following two panels, doublet discrimination was performed; First based on FSC-H and FSC-area (**A**), and second based on SSC–H and SSC-area (**A**). Subsequently, in order to identify the lymphocytes, defined as CD45^high^SSC^low^, the live single cells were shown in a CD45/SSC plot. Next, the CD3^+^ T cells were selected in the lymphocyte gate, and in the final panel the CD4^+^ and CD8^+^ subsets were defined among the CD3^+^ T cells. **B** Panels display the expression of TIGIT, DNAM-1, CTLA-4, and PD-1 in the CD4^+^ T cell subset. **C** Panels depict the expression of the same receptors in the CD8^+^ T cell subset. Red subsets display the results of the FM2 stained sample, which allow for objective gating, while blue subsets show the same sample stained with the nine-color panel

Samples were analyzed immediately after staining on an ACEA NovoCyte Quanteon 4025 flow cytometer (Agilent Technologies, Inc. Santa Clara, CA, USA). Consistency and stability of the instrument were verified on a daily basis using NovoCyte 6 peak QC Particles (Agilent Technologies, Inc.). Compensation was performed using UltraComp eBeads™ (ThermoFisher Scientific) stained with the antibodies and cells stained with 7-AAD. Data were analyzed using FlowJo™ software version 10.7.2 (BD Biosciences). The FM2 controls were used for objective gating of TIGIT, DNAM-1, CTLA-4, and PD-1, and both the percentage of the positive subset and the median fluorescence intensity (MFI) of this positive subset were evaluated. Expression of CD4 and CD8 were analyzed as percentages of the CD3^+^ lymphocytes. The gating strategy is shown in Fig. 1.

### Statistical Analysis

Both the fraction of cells expressing a given marker and the MFI of each marker on CD4 and CD8 T-cells were expressed as medians. For normally distributed data sets (tested by a q-q plot), an unpaired t test was used for analysis of differences between groups. Otherwise, the data sets were tested using the Mann Whitney U test. The selected statistical tests used for each data set are specified in Tables 2 and 3. *p* values of less than 0.05 were considered significant. All statistical analyses were performed using STATA version 16.0 for PC (Stata Corp LP, College Station, TX, USA) and GraphPad Prism version 7.05.

## Results

### Patient Characteristics

A total of 58 MM patients participated in the study: 19 with newly diagnosed treatment-naïve MM (NDMM) and 39 who were progressing on DARA monotherapy or a DARA-containing regimen (DRMM). DRMM had received a median of three prior lines of therapy (Table 1).

**Table 1:** Patient characteristics

Patient group	NDMM *n* = 19	DRMM *n* = 39
Age^a^; years; median (range)	80 (42–86)	68 (47–82)
Gender; *n* (%)		
Female	8 (42)	23 (59)
Male	11 (58)	16 (41)
Immunoglobulin subtype^a^; *n* (%)		
IgG	10 (53)	23 (59)
IgA	6 (32)	3 (8)
Light-chain only	2 (11)	11 (28)
Non secretory	1 (5)	2 (5)
ISS^a^; *n* (%)		
I	4 (22)	16 (41)
II	8 (44)	8 (20)
III	2 (11)	5 (13)
Unknown	4 (22)	10 (26)
ECOG performance status^a^; *n* (%)		
0	9 (47)	23 (59)
1	8 (44)	7 (18)
2	1 (5)	1 (3)
Unknown	1 (5)	8 (21)
Fluorescence in situ hybridization^b^; *n* (%)		
High-risk	2 (11)	9 (23)
Standard-risk	17 (89)	19 (49)
Unknown	0 (0)	11 (28)
Number of prior lines of therapy; median (range)	0 (0–0)	3 (0–17)
Treatment; *n* (%)		
Daratumumab + IMID	NA	14 (36)
Daratumumab + PI	NA	6 (15)
Daratumumab monotherapy	NA	6 (15)
Daratumumab + other	NA	13 (33)

^a^At the time of diagnosis of multiple myeloma

^b^If assessed more than once, the most recent result prior to initiation of daratumumab treatment is shown. High-risk aberrations were defined by the presence of either t(4;14), t(14;16) or del17p, each detected with a cutoff of 10% according to international standards for cytogenetic evaluation

*NDMM* newly diagnosed multiple myeloma patients, *DRMM* daratumumab refractory multiple myeloma patients, *ISS* international staging system, *NA* not applicable, *IMID* immunomodulatory imide drug, *PI* proteasome inhibitor

### Decreased CD4:CD8-Ratio in the BM of DRMM Patients

In the BM, the median CD4:CD8-ratios were 1.10 (range 0.31–14.31) for NDMM and 0.55 (range 0.13–5.78) for DRMM, *p* = 0.0014. When measured in PB, the NDMM had a median CD4:CD8-ratio of 1.26 (range 0.54–24.90), while this ratio in the DRMM was 1.19 (range 0.16–8.10), *p* = 0.07.

### Expression of Checkpoint Molecules by CD4^+^ T-Cells

In order to reveal divergent expression patterns of the receptors CTLA-4, DNAM-1, PD-1, and TIGIT on CD4^+^ T cells from both PB and BM, a comparison between the two groups of MM patients was performed. Both the percentage of the positive subsets and the receptor expression level by MFI, were taken into consideration.

**Fig. 2:**
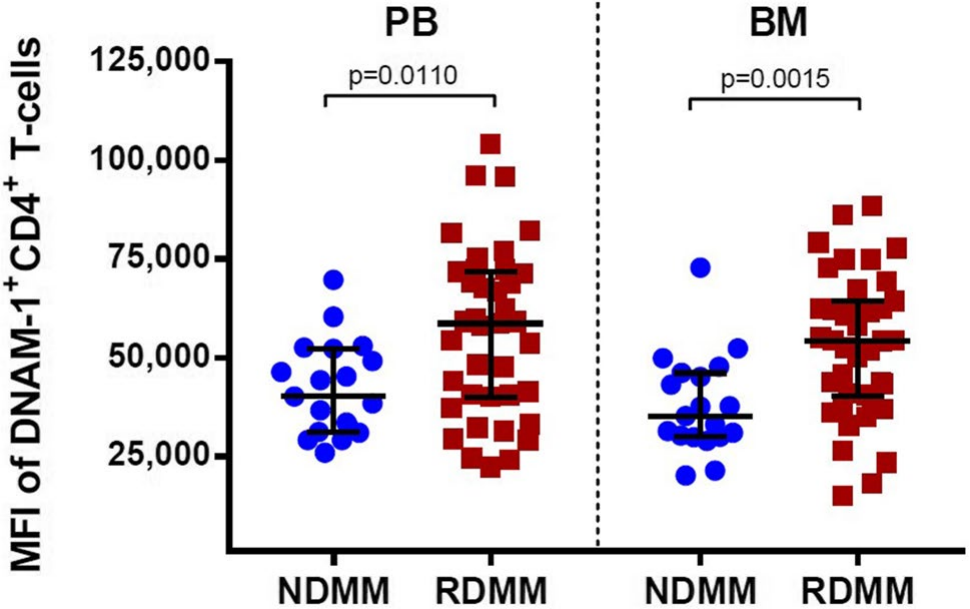
Median fluorescence intensity of DNAM-1 on DNAM-1^+^CD4^+^ T-cells from PB and BM. *PB* peripheral blood; *BM* bone marrow, *NDMM* newly diagnosed multiple myeloma patients; DRMM = daratumumab-refractory multiple myeloma patients

Almost all CD4^+^ T-cells (> 90%) from PB and BM of both groups of patients expressed DNAM-1, but the level of expression (MFI) of DNAM-1 was higher in DRMM compared with NDMM, both in the PB and BM (median MFI: 58,739 versus 40,206, *p* = 0.0110 in PB; 54,257 versus 35,240, *p* = 0.0015 in BM) (Tables 2 and 3, Fig. 2). When comparing NDMM to the six DRMM patients who had received DARA monotherapy, we found the same differences, although non-significant due to the small sample size. The percentage of CD4^+^CTLA-4^+^ T cells was very low in all samples, hence the numbers of acquired positive events were small. Nonetheless, this subset was statistically larger in both PB and BM of DRMM as compared to NDMM (median 0.66% versus 0.48%, *p* = 0.0346 in PB, median: 1.035% versus 0.66%, *p* = 0.0125 in BM) (Table 2). When analyzing the percentages of CD4^+^ T cells expressing either PD-1 or TIGIT in PB and BM samples of NDMM and DRMM, there was no statistical difference (Table 2). Moreover, we did not find any difference between the groups when evaluating the expression level of these receptors, as determined by MFI of PD-1 and TIGIT on the CD4^+^PD-1^+^ T cells and the CD4^+^TIGIT^+^ T-cells, respectively (Table 3).

**Table 2:** Distribution of immune checkpoint receptors on CD4^+^ and CD8 ^+^ T cells in BM and PB of myeloma patients

	PB			BM		
	NDMM median % (range)	DRMM median % (range)	Significant (*p* value)	NDMM median % (range)	DRMM median % (range)	Significant (*p* value)
CD4^+^ T-cells						
DNAM-1	94.1 (84.4–97.6)	95.8 (84.3–99)	Yes (0.0269)	92.6 (86.1–97.9)	95.0 (84.1–99.5)	Yes (0.0125)
CTLA-4	0.48 (0.15–1.24)	0.66 (0.029–2.36)	Yes (0.0315)	0.66 (0.15–2.57)	1.035 (0.19–7.43)	Yes (0.0117)
PD-1	19.3 (5.35–66.2)	26.0 (3.67–84.4)	No (0.2503)	18.7 (4.95–54.6)	30.2 (2.22–80.9)	No (0.0857)
TIGIT	18.6 (8.34–28.7)	19.8 (4.28–53)	No (0.4516)	17.8 (7.1–31.1)	19.2 (3.74–49.1)	No (0.5242)
CD8^+^ T-cells						
DNAM-1	81.1 (55.1–95.5)	86.4 (54.7–97.7)	No (0.2975)	74.9 (47.7–95.1)	78.7 (49.6–97.4)	No (0.8470)
CTLA-4	0.19 (0.028–0.76)	0.18 (0.041–1.05)	No (0.9182)	0.26 (0.076–1.01)	0.185 (0.055–1.73)	No (0.2400)
PD-1	13.8 (2.6–62.6)	14.8 (2.91–80.3)	No (0.8858)	21.5 (4.04–60.04)	18.1 (1.6–86.7)	No (0.8406)
TIGIT	80.6 (57.1–94.8)	80.5 (47.9–94.5)	No (0.5962)	84.2 (65.6–95.3)	81.5 (52.8–95.3)	No (0.2975)

*PB* peripheral blood, *BM* bone marrow, *NDMM* newly diagnosed multiple myeloma patients, *DRMM* daratumumab refractory multiple myeloma patients

Flow cytometric analysis of immune receptor molecules are presented as median percentage of cells expressing the respective molecules. Statistical significance was tested using Mann Whitney *U* test for all datasets

**Table 3:** MFI of immune checkpoint receptors on receptor positive subset of CD4^+^ and CD8^+^ T cells in myeloma patients

	PB			BM		
	NDMM median MFI (range)	DRMM median MFI (range)	Significant (*p* value)	NDMM median MFI (range)	DRMM median MFI (range)	Significant (*p* value)
DNAM-1^+^CD4^+^ T-cells						
DNAM-1	40,206 (25,970–69,672)	58,739 (22,384–104,050)	Yes (0.0110)*	35,240 (20,129–72,760)	54,257 (15,114–88,388)	Yes (0.0015)*
CTLA-4^+^CD4^+^ T-cells						
CTLA-4	1124 (901–1364)	1157 (842–2361)	No (0.3172)*	1073 (807–1403)	1130 (857–2515)	No (0.3889)*
PD-1^+^CD4^+^ T-cells						
PD-1	3555 (2336–5641)	3411 (1796–10,656)	No (0.5849)*	3166 (2362–5523)	3477 (1878–11,512)	No (0.5404)*
TIGIT^+^CD4^+^ T-cells						
TIGIT	22,573 (19,239–33,453)	24,938 (18,666–45,653)	No (0.2271)*	21,414 (15,328–26,841)	22,390 (12,385–42,418)	No (0.3503)*
DNAM-1^+^CD8^+^ T-cells						
DNAM-1	32,891 (15,768–48,567)	33,407 (17,281–54,751)	No (0.9015)^#^	28,172 (12,408–50,468)	27,497 (12,829–47,270)	No (0.8470)^#^
CTLA-4^+^CD8^+^ T-cells						
CTLA-4	1039 (914–1382)	1083 (803–1648)	No (0.1336)*	1055 (815–1268)	1072 (876–1989)	No (0.4521)*
PD-1^+^CD8^+^ T-cells						
PD-1	4486 (3405–6774)	4371 (2503–7687)	No (0.7923)^#^	4968 (3319–6662)	4535 (3223–7938)	No (0.9406)^#^
TIGIT^+^CD8^+^ T-cells						
TIGIT	28,485 (16,677–66,034)	27,173 (15,670–44,422)	No (0.2751)*	30,611 (18,442–54,170)	27,824 (15,607–45,270)	No (0.1826)*

*PB* peripheral blood, *BM* bone marrow, *NDMM* newly diagnosed multiple myeloma patients, *DRMM* daratumumab refractory multiple myeloma patients

Median MFI of immune checkpoint receptors on DNAM-1^+^/CTLA-4^+^/PD-1^+^/TIGIT+ subset of CD4^+^ and CD8^+^ T-cells

Statistical significance was tested using either Mann Whitney *U* test = * or *t* test = #

When looking only at the NDMM, we found that the MFI of PD-1 (median MFI: 4060 versus 3006, *p* = 0.002) and TIGIT (median MFI: 23,235 versus 21,008, *p* = 0.040) on CD4^+^ T-cells from the BM was higher in patients with a poor response to the first line of treatment (*n* = 4) compared to patients with a partial or better response (*n* = 10) according to the IMWG guidelines [19]. Furthermore, the percentage of TIGIT^+^CD4^+^ T-cells in PB was higher in patients with a poor response (median 24.2% versus 15.1%, *p* = 0.036). None of the patients received DARA in their first line of treatment. Due to the sample size, these data need to be interpreted with caution.

### Expression of Checkpoint Molecules by CD8^+^ T-Cells

Similar to the CD4^+^ T-cells, the majority of CD8^+^ T-cells (70–80%) expressed DNAM-1 (Table 2). In contrast to CD4^+^ T-cells, there was no difference in the MFI of CD8^+^DNAM-1^+^ in PB and BM between the NDMM and DRMM groups (median 32,891 versus 33,407, *p* = 0.9015 in PB; median 28,172 versus 27,497, *p* = 0.8377 in BM) (Table 3). Like the CD4^+^ T-cells, the percentage of CD8^+^CTLA-4^+^ T-cells was very low, and the median MFI of CTLA-4 on the CD8^+^CTLA-4^+^ T-cells was also low (median 1039 versus 1083, *p* = 0.1316 in PB; median 1055 versus 1072, *p* = 0.4462 in BM) (Table 3). As for CD4^+^ T-cells, the percentages of CD8^+^ T cells expressing either PD-1 or TIGIT in PB and BM samples of NDMM and DRMM were not statistically different (Table 2). Furthermore, we did not find any difference between the groups when analyzing the expression level of these receptors as determined by the MFI of PD-1 and TIGIT on the CD8^+^PD-1^+^ T cells and the CD8^+^TIGIT^+^ T-cells, respectively (Table 3).

There was no correlation between the expression of checkpoint molecules by CD8^+^ T-cells and the response to treatment for NDMM.

### TIGIT is the Most Frequently Expressed Immune Checkpoint Receptor on BM CD8^+^ T-Cells

**Fig. 3:**
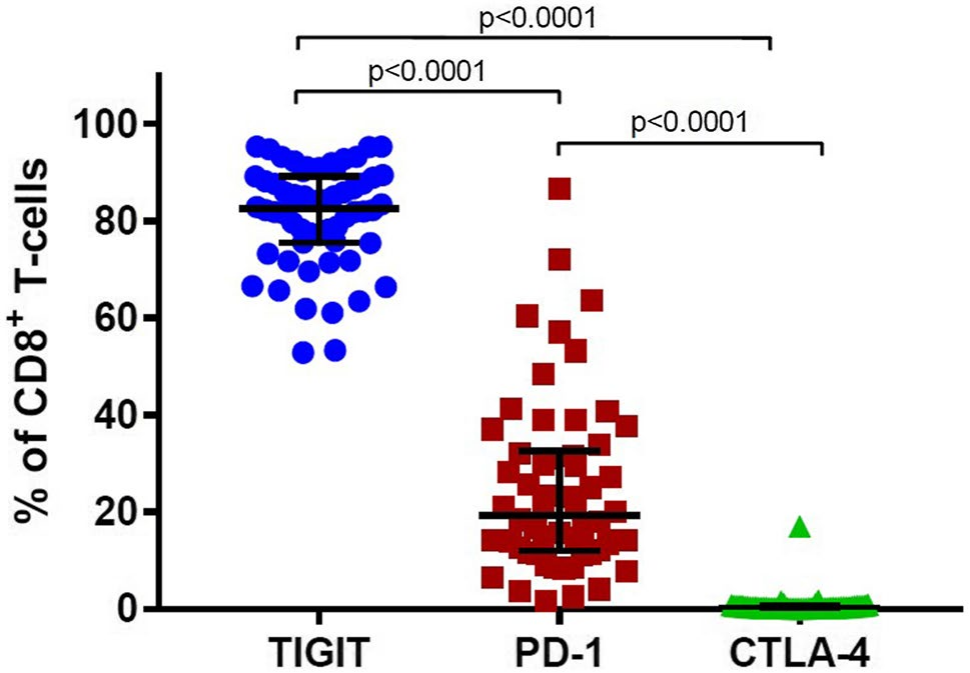
The expression of inhibitory immune checkpoint receptors on CD8^+^ T-cells from all patients (*n* = 58)

TIGIT was the most frequently expressed checkpoint receptor on the BM CD8^+^ T-cells. It was present on significantly more cells than PD-1 (median 82.5% versus 19.25%, *p* < 0.00001) (Fig. 3). Of the six patients with PD-1 expressed on more than 50% of the BM CD8^+^ T-cells, one had received no prior treatment, two had received DARA and an immunomodulatory drug, and three had received DARA in other combinations. CTLA-4 was expressed on less than 1% of BM CD8^+^ T-cells (Fig. 3). For BM CD4^+^ T-cells, more cells expressed PD-1 than TIGIT (median 26.7% versus 18.4%, *p* = 0.0131), but the fractions of BM CD4^+^ T-cells expressing either of these checkpoint receptors were much lower than the percentage of TIGIT-expressing BM CD8^+^ T-cells.

## Discussion

The immune regulatory receptors DNAM-1, CTLA-4, PD-1 and TIGIT are expressed by T-cells, and interact with their ligands upon T-cell receptor ligation, resulting in negative or positive regulation of T-cell activity. Here, we examined the presence of these checkpoint molecules on T-cell subsets from NDMM and DRMM, in order to identify changes that correlated with resistance to treatment with DARA.

In this study, we have demonstrated an increased expression of DNAM-1 by CD4^+^ T-cells from DRMM compared to NDMM. The biological and possible clinical significance of the finding is not known. Allegedly, DNAM-1 is upregulated on activated CD4^+^ and CD8^+^ T-cells. It competes with TIGIT for the CD155 receptor found on antigen-presenting cells, tumor cells and virus-infected cells [22]. Specifically, CD155 is expressed on most MM cells [9, 10]. We found that both the percentage of DNAM-1^+^CD4^+^ T-cells and the MFI of DNAM-1 on DNAM-1^+^CD4^+^ T-cells in PB and BM of DRMM were higher compared to NDMM. Krejcik et al. found that a subpopulation of CD38^+^CD4^+^ T regulatory cells (Tregs) that demonstrated a suppressive capacity against cytotoxic T-cells was eliminated by treatment with DARA due to their expression of CD38 [12]. This process correlated with recruitment and activation of CD8^+^ T-cells and the clinical response to DARA. In our study, almost all CD4^+^ T-cells expressed DNAM-1, so we are evidently not dealing with a subpopulation of CD4^+^ Tregs, as these occur in low numbers. To investigate the role of DNAM-1^+^ CD4^+^ T-cells in the development of resistance to DARA, an in-depth subset analysis of the CD4^+^ T-cells and functional studies are needed. In this context, it could be informative to add DARA-treated patients in complete response to the analyses.

We did not observe any differences between NDMM and DRMM with regards to the expression of PD-1. A few patients expressed PD-1 on more than 50% of their BM CD8^+^ T-cells. These patients had received different DARA-containing regimens. This is in contrast with a number of studies that examined the expression of PD-1 on T-cells from myeloma patients at different stages of the disease. Minnie et al. analyzed T-cells isolated from the BM of mice with MM and found that PD-1 was overexpressed on CD8^+^ T-cells from mice with relapsed MM (relapse/refractory MM; RRMM) compared to mice with MM in remission [23]. Furthermore, Zelle-Rieser et al. reported that the expression of PD-1 on CD4^+^ and CD8^+^ T cells isolated from the BM of RRMM patients was higher compared to T-cells isolated from the BM of healthy donors [5]. One study, with a design very similar to ours, found that the median percentage of CD8^+^ T-cells expressing PD-1 was higher in the BM of RRMM compared to that in NDMM (35% and 28%, respectively) [24]. Although we were unable to show such differences, we did observe percentages of PD-1 expressing T cell subsets in approximately the same range as reported by Paiva and colleagues (Table 2). The discrepancy between the two studies could be due to the composition of the patient groups analyzed: while the DRMM group in the present study was uniform, consisting only of patients relapsing on DARA, the treatment regimen of the equivalent group in the Paiva study was not described. However, as the FDA did not approve daratumumab until November 2015, probably the group did not hold patients treated with this drug. Thus, from the information available, it seems plausible that there may be a difference in the expression of PD-1 when comparing RRMM with either MM patients in remission or healthy donors, but whether there is a difference when comparing NDMM to RRMM is not yet clarified.

Several clinical trials have investigated the use of PD-1 or PD-L1 inhibitors in MM. Monotherapy with PD-1 inhibitors did not induce clinical response in MM [7, 8]. Furthermore, phase 3 studies, which evaluated the combination of a PD-1 inhibitor with immunomodulatory drugs (IMIDs) were stopped prematurely, due to an increased frequency of serious adverse events in the experimental arm [25, 26]. In a phase 2 clinical trial, the PD-L1 inhibitor durvalumab was used as an add-on to DARA, for patients who recently progressed on a DARA-containing treatment, in an attempt to reverse the resistance to DARA [27]. None of the 18 patients treated in the trial obtained a partial response or better. Interestingly, the investigators compared PD-1 expression on the patients’ T cells at baseline (i.e. the time point of progression on daratumumab) and after 6 weeks of treatment with durvalumab and DARA, but found no difference in expression. These data are in line with our results, indicating that the PD-1—PD-L1 axis may not be the key signaling pathway driving the development of resistance to daratumumab.

Like for PD-1, we found no difference in the expression of TIGIT when comparing NDMM to DRMM, but observed that TIGIT was the inhibitory receptor most frequently expressed by CD8^+^ T cells. In contrast to our findings, Minnie and colleagues reported that the frequency of TIGIT expression was higher on CD8^+^ T-cells from mice with relapsed MM compared to MM in remission or healthy mice [23]. Nevertheless, studies in humans have demonstrated that compared to PD-1 and CTLA-4, TIGIT was more frequently expressed on CD8^+^ T cells from the MM patients thus concurring with our data [11, 28]. Collectively, this suggests that TIGIT may play a major role as an immune regulator of cytotoxicity. The position as a receptor of importance is supported by studies showing that MM mice treated with anti-TIGIT mAbs lived significantly longer [11, 23]. In further support of the importance of TIGIT in MM, Neri et al. found upregulation of TIGIT on T-cells from DARA non-responders compared with DARA responders [29]. In the BM of DRMM patients, we found an excess of CD8^+^ T-cells, but despite of their presence, the disease was not controlled, maybe because of checkpoint inhibition most likely mediated by TIGIT. Anti-TIGIT mAbs in MM are in currently recruiting clinical trials, both as monotherapy and in combination with anti-MM drugs.

CTLA-4 was expressed at a low level by the T-cells analyzed, and even though there was a significant difference in its expression when comparing NDMM to DRMM, we do not consider this difference to be clinically significant. Other studies confirm our observations [11, 27]. A combination of the PD-1 inhibitor nivolumab and the CTLA-4 inhibitor ipilimumab was tested in vivo in 7 MM patients. None of these patients had any objective response [30]. This supports our interpretation that CTLA-4 is not a key immune regulatory receptor in MM.

Multiple checkpoint molecules exist besides the four which we examined here. High expression of the checkpoint receptor CD200 in AML is associated with poor overall survival, but treatment with an anti-CD200 antibody can restore the AML immune response [31, 32]. Pochard et al. found that low expression of CD200 in MM patients was associated with a good response to DARA [33]. Future combination studies of checkpoint inhibitors are required to clarify if these observations will be clinically important.

The patients in the DRMM group were included when they progressed on DARA with or without other anti-MM drugs. It is a limitation of this study that the DRMM group is not totally uniform, but it does reflect the real world situation where DARA is administered in different combinations. When we examined the findings regarding DNAM-1 expression on CD4^+^ T-cells in the subpopulation of patients receiving monotherapy with DARA to the rest of the DARA-exposed population, we found no obvious difference. We chose NDMM as a control group to avoid the impact of other drugs given before DARA on the BM microenvironment. Brauneck et al. found that CD8^+^ T-cells from NDMM showed an increased expression of PD-1 and TIGIT compared to healthy controls [34]. As we did not include a group of healthy controls, we were unable to perform the same comparison; however, we found no correlation between the expression of checkpoint molecules on CD8^+^ T-cells and response to first line treatment. In contrast, our small dataset on this group of untreated patients may indicate that the expression of PD-1 and TIGIT on CD4^+^ T-cells could play a role. Further studies with larger sample sizes are needed. Note that none of the NDMM patients received DARA in the first line of treatment, but all were eligible for DARA in their second line of treatment, as recommended by local guidelines in Denmark.

## Conclusions

In conclusion, our study supports the hypothesis that TIGIT may play a more important role as an immune checkpoint in MM than PD-1 and CTLA-4, and that anti-TIGIT mAbs could be effective in MM. Whether or not the DNAM-1^+^CD4^+^ T-cells play a role in the development of resistance to DARA needs further exploration and characterization of the involved T-cells.

## Data Availability

The data presented in this study are available on request from the corresponding author.
